# *GDI2* is a novel diagnostic and prognostic biomarker in hepatocellular carcinoma

**DOI:** 10.18632/aging.203748

**Published:** 2021-12-11

**Authors:** Wen Zhang, Zhongjian Liu, Shilin Xia, Lei Yao, Lan Li, Ziying Gan, Hui Tang, Qiang Guo, Xinmin Yan, Zhiwei Sun

**Affiliations:** 1 School of Medicine, Kunming University of Science and Technology, Affiliated by The First People’s Hospital of Yunnan Province, Kunming 650504, Yunnan, China; 2 Yunnan Digestive Endoscopy Clinical Medical Center, Gastroenterology Department, The First People’s Hospital of Yunnan Province, The Affiliated Hospital of Kunming University of Science and Technology, Kunming 650032, Yunnan, China; 3 Clinical Laboratory of Integrative Medicine, The First Affiliated Hospital of Dalian Medical University, Dalian 116011, Liaoning, China; 4 General Surgery Department, The Second Affiliated Hospital of Harbin Medical University, Harbin 150086, Heilongjiang, China; 5 Ophthalmology Department, Jiangxi Provincial People’s Hospital, Nanchang 330006, Jiangxi, China

**Keywords:** *GDI2*, HCC, prognosis, biomarker, TCGA

## Abstract

Background: GDP Dissociation inhibitor 2 (*GDI2*) gene has been correlated with some important biological processes in a variety of cancers, whereas the role of *GDI2* in hepatocellular carcinoma (HCC) is ill-defined. We aimed to demonstrate the relationship between *GDI2* and HCC based on The Cancer Genome Atlas (TCGA) data mining.

Methods: The expression of *GDI2* was compared between cancer and normal tissues of 371 HCC patients collected from TCGA-LIHC, and verified in HCC cell lines. Gene set enrichment analysis (GSEA) was applied to annotate biological function of *GDI2*. Furthermore, Wilcoxon rank sum test, Logistics regression, as well as Cox regression and Kaplan-Meier survival analysis, were employed to evaluate the association of *GDI2* expression with clinicopathological characteristics, and survival status of HCC patients, respectively.

Results: It showed that the expression of *GDI2* was much higher in tumor tissues than in normal tissues (*P* < 0.001) of HCC patients. And the elevated expression of *GDI2* was correlated with more aggressive HCC tumor status, including severe primary tumor extent, advanced pathological stage, serious histologic grade, and mutated TP53 status (*P* < 0.05). Moreover, high *GDI2* expression was strongly associated with a poor survival rate (*P* < 0.001). Both enrichment and immune infiltration analyses implied that *GDI2*-associated signaling mainly involve lipid metabolism and extracellular matrix (ECM) constructing pathways related to tumor microenvironment (TME) (*P* < 0.05).

Conclusions: The elevated expression of *GDI2* predicts poor prognosis in HCC patients, indicating that *GDI2* could be applied as a predictive biomarker for diagnosis and prognosis of HCC.

## INTRODUCTION

The major histological type of primary liver cancer (PLC) is hepatocellular carcinoma (HCC), which seriously threatens human health [1] and is the fifth most common malignant tumor covering 700, 000 newly diagnosed cases globally every year [2]. Though the development of surgery and various drug therapies has significantly increased the survival rate of patients with early HCC [3], the overall five-year survival rate of advanced HCC patients has not been exceeding 5% [4]. Moreover, as the pathogenesis of HCC is extremely complex with complicated interactions involving multiple genes at multiple steps [5], most cases of HCC are already at advanced stages at diagnosis due to rapid progression resulting from limited understanding of its mechanism. In addition to high rate of recurrence and metastasis, unsatisfactory efficacy of existing targeted drugs, and complexity of anti-HCC drug resistance [6], the lack of biomarkers that are specific for tumor types or disease stages represents a critical gap in the current understanding and treatment of HCC [7–9]. In particular, HCC is considered a virus-related malignancy in which hepatitis B and C viruses (HBV and HCV) are major etiological factors [10]; while nonalcoholic fatty liver disease (NAFLD) also becomes the fastest growing cause of HCC globally [11]. Both trends are rather important because the gene signature of HCC could be altered by the shifting of etiology mediated by either virus infection or fatty metabolism. Therefore, it is crucial to discover new diagnostic and therapeutic targets for profound HCC research and effective HCC treatment. Considering that researchers have gained valuable evidence from genetic studies [12], it may be possible to identify crucial biomarkers underpinning HCC pathogenesis or therapeutic targets for HCC treatment by screening gene networks for changes related to tumor formation and progression.

GDP dissociation inhibitor 2 (*GDI2*, Gene ID:2665), located in region from 5,765,223 to 5,842,132 of the reverse strand on Chromosome 10, is a family member of GDP dissociation inhibitors (GDIs) [13]. *GDI2* is a ubiquitously expressed gene that encodes proteins to regulate the GDP-GTP exchange reaction of members from the Rab family, or small GTP-binding proteins from the Ras superfamily, and is involved in vesicular trafficking of molecules between cellular organelles. GDIs slow the rate of dissociation of GDP from rab proteins and release GDP from membrane-bound rabs [14].

As a regulator of GDP-GTP exchange reaction, *GDI2* was reported to participate in various biological processes of solid tumors, such as breast cancer (BC) [15], pancreatic carcinoma (PC) [16], gastric cancer (GC) [17], and so on. However, the relationship between *GDI2* and HCC has not been reported yet. The only report studied in hepatic carcinoma cells HepG2 was to demonstrate that the methanol extract of T. indica fruit pulp altered the release of *GDI2* from HepG2 cells, which possibly correlated *GDI2* gene to cellular lipid metabolism [18]. Hence, we performed advanced bioinfomatics analyses on the expression and clinical association of *GDI2* gene in LIHC project from The Cancer Genome Atlas (TCGA) [19] in order to explore the role of *GDI2* in HCC. Our results figured out that the *GDI2* could be applied as a potential biomarker for diagnosis and prognosis for HCC patients, thus providing novel target and strategies for HCC treatment.

## RESULTS

### Clinical characteristics

The clinical characteristics of 371 hepatocellular carcinoma (HCC) patients from TCGA were collected as shown in Table 1, including gender, race, TNM stage, pathologic stage, vascular invasion and tumor status, as well as HCC-specific index as adjacent hepatic tissue inflammation, fibrosis ishak score and Child-Pugh grade. In present study, a total of 121 female patients (54.2%) and 250 male (54.2%) patients were analyzed, including 184 white patients (49.6%) and 175 non-white patients (47.2%; 158 Asian patients and 17 Black or African American patients). The tumor status involved 201 tumor free (54.2%) cases and 151 cases with tumor (40.7%), while 109 with (19.4%) and 206 without (55.6%) vascular invasion. Stage I disease was found in 171 patients (46.1%), and Stage II, III, IV in 85 (22.9%), 85 (22.9%), and 5 (1.3%) patients, respectively. Most tumors were distributed among 48.8% T1 Stage (n=181), 25.3% T2 (n=94), 21.6% T3 (n=80), and 3.5% T4 (n=13). As not all the HCC patients could offer intact clinical data, the censoring values made the total number fail to sum up to 371 cases for each clinical count.

**Table 1:** Clinical characteristics of HCC patients.

**Characters**	**Level**	**Low expression of *GDI2***	**High expression of *GDI2***	***P* value**
Number		186	185	
T stage (%)	T1	100 (53.8%)	81 (44.5%)	0.163
	T2	47 (25.3%)	47 (25.8%)	
	T3	35 (18.8%)	45 (24.7%)	
	T4	4 (2.2%)	9 (4.9%)	
N stage (%)	N0	117 (99.2%)	135 (97.8%)	0.627
	N1	1 (0.8%)	3 (2.2%)	
M stage (%)	M0	129 (98.5%)	137 (98.6%)	1.000
	M1	2 (1.5%)	2 (1.4%)	
Pathologic stage (%)	Stage I	94 (53.4%)	77 (45.0%)	0.138
	Stage II	45 (25.6%)	41 (24.0%)	
	Stage III	34 (19.3%)	51 (29.8%)	
	Stage IV	3 (1.7%)	2 (1.2%)	
Residual tumor (%)	R0	169 (95.5%)	155 (93.9%)	0.458
	R1	7 (4.0%)	10 (6.1%)	
	R2	1 (0.6%)	0 (0.0%)	
Histologic grade (%)	G1	30 (16.2%)	25 (13.8%)	0.214
	G2	97 (52.4%)	80 (44.2%)	
	G3	53 (28.6%)	69 (38.1%)	
	G4	5 (2.7%)	7 (3.9%)	
Gender (%)	Female	53 (28.5%)	68 (36.8%)	0.113
	Male	133 (71.5%)	117 (63.2%)	
Race (%)	Asian	75 (42.1%)	83 (45.9%)	0.628
	Black or African American	10 (5.6%)	7 (3.9%)	
	White	93 (52.2%)	91 (50.3%)	
Adjacent hepatic tissue inflammation (%)	Mild	48 (38.4%)	51 (46.8%)	0.351
	None	68 (54.4%)	49 (45.0%)	
	Severe	9 (7.2%)	9 (8.3%)	
Child-Pugh grade (%)	A	117 (93.6%)	100 (87.7%)	0.108
	B	7 (5.6%)	14 (12.3%)	
	C	1 (0.8%)	0 (0.0%)	
Fibrosis ishak score (%)	0	44 (39.3%)	30 (30.0%)	0.242
	1/2	13 (11.6%)	18 (18.0%)	
	3/4	17 (15.2%)	11 (11.0%)	
	5/6	38 (33.9%)	41 (41.0%)	
Vascular invasion (%)	No	107 (67.3%)	99 (63.5%)	0.551
	Yes	52 (32.7%)	57 (36.5%)	
Tumor status (%)	Tumor free	107 (59.8%)	94 (54.3%)	0.356
	With tumor	72 (40.2%)	79 (45.7%)	
TP53 status (%)	Mut	43 (24.3%)	59 (32.6%)	0.105
	WT	134 (75.7%)	122 (67.4%)	
Age (median [IQR])		62.50 [54.00, 69.00]	59.00 [50.75, 68.00]	0.073
Height (median [IQR])		168.00 [160.00, 174.00]	168.00[163.00, 174.00]	0.687
Weight (median [IQR])		72.00 [61.00, 87.50]	68.00 [58.00, 79.00]	0.035*
BMI (median [IQR])		25.15 [22.23, 29.67]	23.88 [21.21, 27.47]	0.020*
AFP(ng/ml) (median [IQR])		10.00 [3.00, 150.75]	24.00 [5.00, 548.25]	0.019*
Albumin(g/dl) (median [IQR])		4.00 [3.50, 4.30]	4.00 [3.50, 4.30]	0.815
Prothrombin time (median [IQR])		1.10 [1.00, 9.75]	1.10 [1.00, 5.25]	0.173

^*^P < 0.05; ^**^ *P* < 0.01.

### Identification of differentially expressed genes (DEGs) in HCC

**Figure 1:**
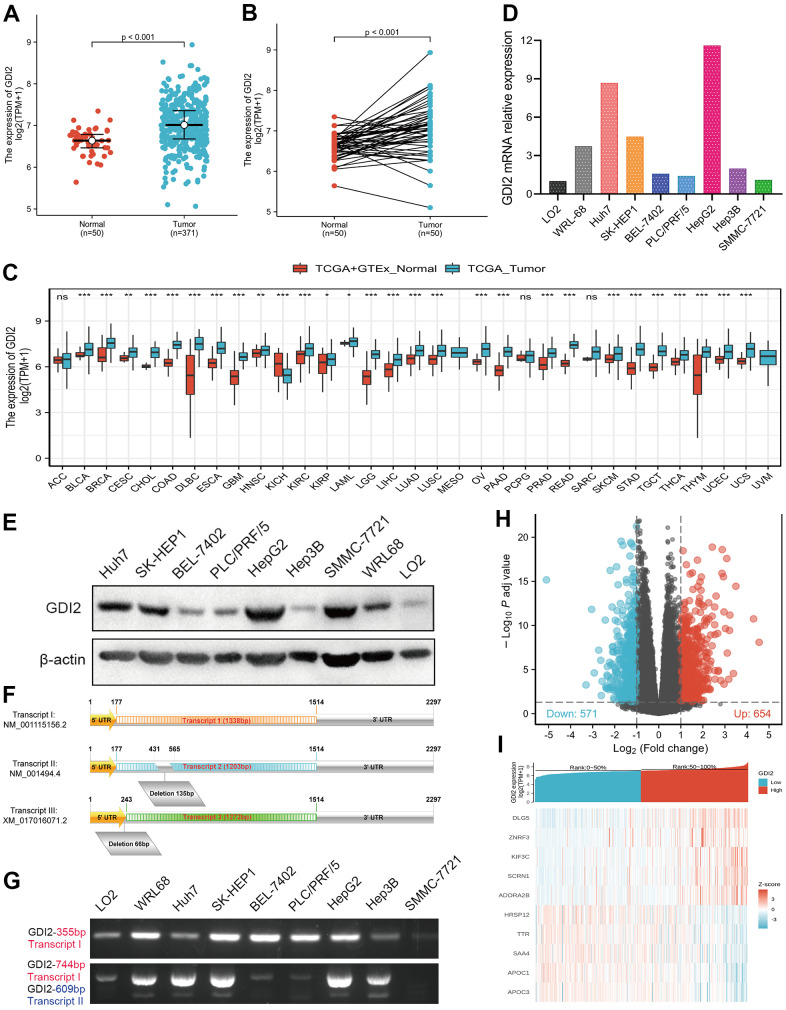
**Differentially expressed genes (DEGs) involved in *GDI2* expressing cancer groups.** (**A**) Elevated expression of *GDI2* between Normal and Tumor tissues of HCC patients. (**B**) Elevated expression of *GDI2* in 50 paired Normal-and-Tumor tissues of HCC patients. (**C**) Box plot of the differentially expression of *GDI2* gene among 33 kinds of pan-cancers. The X-axis represents the pan-cancer types, while the Y-axis denotes the expression of *GDI2*. (**D**) qRT-PCR assay confirmed the *GDI2* mRNA expressions in two normal hepatic cells and seven hepatoma cells. (**E**) Western blot assay confirmed the protein levels of *GDI2* in two normal hepatic cells and seven hepatoma cells. (**F**) Structural diagram of Transcript I, II and III of *GDI2* gene. (**G**) Electrophoretogram for expressions of Transcript I, II and III of *GDI2* gene based on different fragments of PCR primers in two normal hepatic cells and seven hepatoma cells. (**H**) Volcano plot of differential gene profiles between *GDI2*-high and -low groups. In 1225 DEGs, 654 upregulated and 571 downregulated genes were represented by red and blue tones, respectively. (**I**) Heat map of 10 significantly differentially expressed DEGs between *GDI2*-high and -low groups. Normalized expression levels are shown in descending order from red to blue. *P < 0.05, **P < 0.01, ***P < 0.001.

To elucidate whether *GDI2* was positively correlated with HCC occurrence, the *GDI2* expressions were compared between 371 HCC cases and 50 normal tissue cases via RNAseq TMP data from TCGA combined with GTEx, which showed much higher expression of *GDI2* in tumor cases than in normal cases (*P* < 0.001, Figure 1A). Among them, 50 tumor-and-adjacent paired samples were also showed high expression of *GDI2* in tumor compared with paired normal tissues (*P* < 0.001, Figure 1B). To further define the oncogenicity of *GDI2*, comparison of *GDI2* expression between tumor and normal specimens was also made in pan-cancers from TCGA-GTEx database. It was shown that *GDI2* significantly expressed in most of the 33 kinds of solid tumors, such as hepatocellular carcinoma (LIHC, HCC), lung adenocarcinoma (LUAD), cholangiocarcinoma (CHOL), breast cancer (BRCA), glioblastoma (GBM), endometrial carcinoma of uterus (UCEC), etc (Figure 1C). Then the differential expression of *GDI2* was verified between two normal hepatic cell lines (L02, WRL-68) and seven hepatoma cell lines (Huh7, SK-HEP1, BEL-7402, PLC/PRF/5, SMMC-7721, HepG2, Hep3B) *in vitro*. Moreover, which transcript exerts effect in these HCC cell lines was also confirmed by designing different primers amplifying certain fragments for different transcripts of *GDI2* gene. Both qRT-PCR and western blot assays confirmed that compared with normal hepatic cells L02, *GDI2* expression was increased in most of hepatoma cells (Figure 1D, 1E). Although there are three transcripts *GDI2* mRNA, Transcript I (NM_001115156.2) and Transcript II (NM_001494.4) are two confirmed mature transcripts, while Transcript III (XM_017016071.2) is a predictive transcript overlapped with Transcript I only with a latter Start Codon (Figure 1F). Correspondingly, PCR amplification displayed that almost both Transcript I and II of *GDI2* gene contributed to *GDI2* expression in HCC cell lines (Figure 1G). Finally, based on the cut-off criteria (|log2-fold change (FC)|>1, adjusted P-value<0.05) for median value of *GDI2* expression, a total of 1225 Differentially Expressed Genes (DEGs) were identified after the analyses of TCGA RNA-seq data between *GDI2*-high and -low groups, including 654 upregulated and 571 downregulated DEGs illustrated by Volcano Map (Figure 1H), 10 of which were specifically displayed by Heat Map (Figure 1I).

### Functional enrichment analysis associated with *GDI2* by GO, KEGG and GSEA

**Figure 2:**
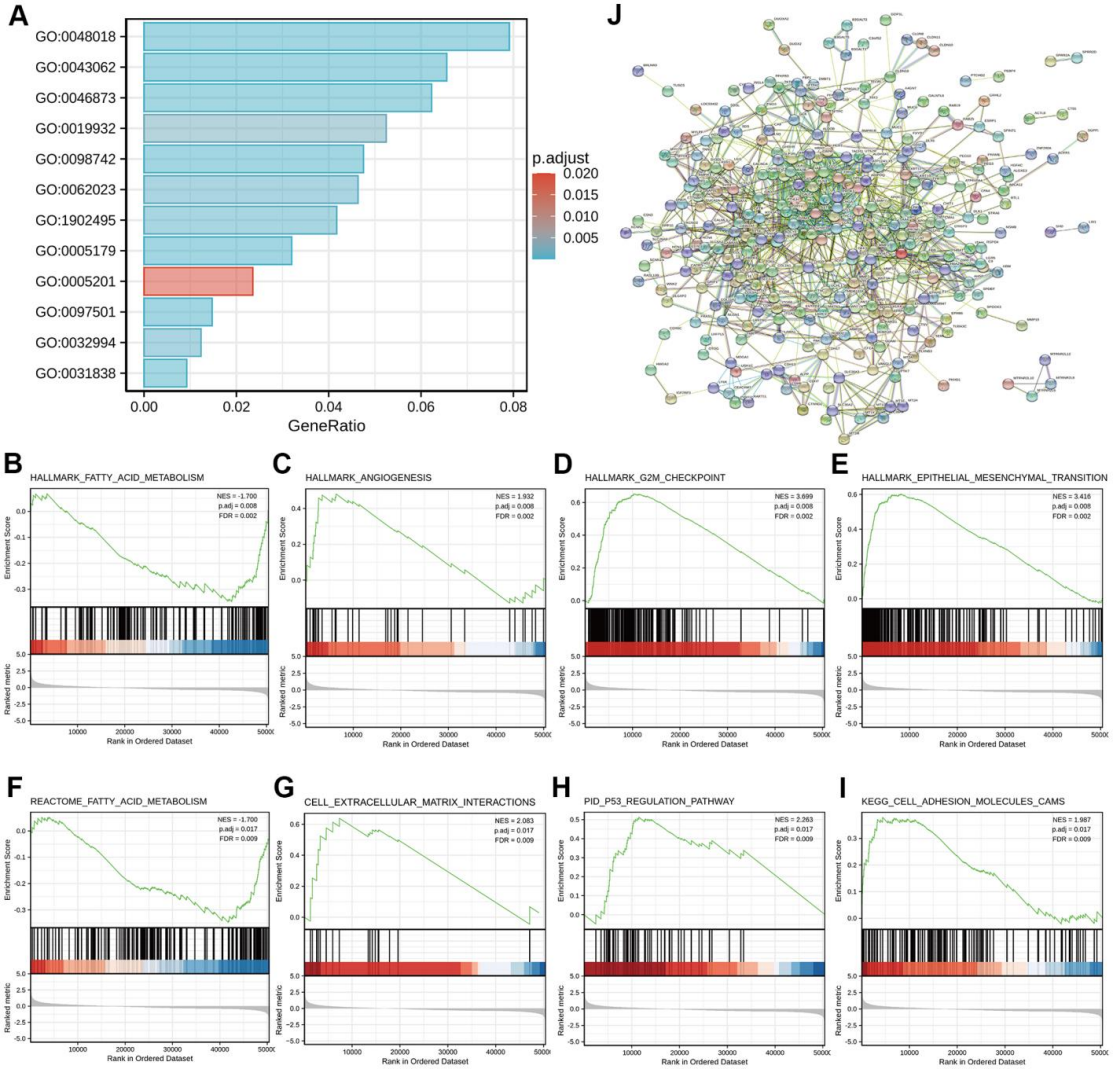
**Functional enrichment analyses of DEGs between high- and low-*GDI2* expression in TCGA-LIHC patients.** (**A**) Twelve significant GO terms and KEGG pathway enrichment of DEGs between high- and low-*GDI2* expression in TCGA-LIHC patients. (**B**–**E**) Enrichment plots of (**B**) Fatty acid metabolism, (**C**) Angiogenesis, (**D**) G2M checkpoint, (**E**) Epithelial mesenchymal transition pathways of Hallmarks symbols by GSEA analysis. ^*^adj.*P* = 0.008, FDR = 0.002. (**F**–**I**) Enrichment plots of (**F**) Fatty acid metabolism by REACTOME, (**G**) Cell extracellular matrix interactions by REACTOME, (**H**) *P53* regulation pathway by PID, (**I**) Cell adhesion molecules CAMS by KEGG of C2_curated symbols by GSEA analysis. adj. ^**^adj.*P* = 0.017, FDR = 0.009. (**J**) Visualized protein-protein interaction enrichment of DEGs associated with *GDI2* expression by STRING online database (Combined score > 0.7).

To better understand the functional implications of *GDI2* in HCC, GO and KEGG functional enrichment analyses were performed based on 1225 DEGs between high- and low-*GDI2* expression, which indicated that the *GDI2*-associated genes engaged in 342 GO terms of biological processes (BP), 62 terms of cellular components (CC) and 74 terms of molecular function (MF). Thereinto, massive enrichment of Receptor ligand activity (GO:0048018), Hormone activity (GO:0005179), Metal ion transmembrane transporter activity (GO:0046873) and Extracellular matrix structural constituent (GO:0005201) categorized by MF, Stress response to metal ion (GO:0097501), Extracellular structure organization (GO:0043062), Cell-cell adhesion via plasma-membrane adhesion molecules (GO:0098742) and Second-messenger-mediated signaling (GO:0019932) categorized by BP, Haptoglobin-hemoglobin complex (GO:0031838), Transmembrane transporter complex (GO:1902495), Protein-lipid complex (GO:0032994) and Collagen-containing extracellular matrix (GO:0062023) categorized by CC, were highly associated with the aberrant expression of *GDI2* (**P < 0.01; Figure 2A).

As for advanced analysis, gene set enrichment analysis (GSEA) was applied to confirm the key signaling pathways referring to *GDI2* expression data sets. Significant differences with normalized enrichment score |NES| > 1.0, adjusted P-value < 0.05, false discovery rate (FDR) < 0.25 in enrichment of the MSigDB Collection h.all.v7.0.symbols [Hallmarks] and c2.cp.v7.0.symbols [curated] were observed in a total of 39 and 690 pathways, respectively. In particular, *GDI2* was related to the Fatty acid metabolism, Angiogenesis, G2M checkpoint, Epithelial mesenchymal transition of Hallmarks symbols (adj.*P* = 0.008, FDR = 0.002; Figure 2B–2E), and similarly, to the Fatty acid metabolism by REACTOME, Cell extracellular matrix interactions by REACTOME, *P*53 regulation pathway by PID, Cell adhesion molecules CAMS by KEGG of C2_curated symbols (adj. *P* = 0.017, FDR = 0.009; Figure 2F–2I).

Furthermore, protein-protein interaction (PPI) enrichment analysis was performed to predict co-regulatory protein network of *GDI2*, and the functional interactions among proteins were illustrated by STRING online database. The interactions with a combined score > 0.7 were considered statistically significant, covering 620 protein network interactions. The resultant protein network illustrated by STRING encompassed the Red Node *GDI2* with interactive proteins as multicolor Bubbles, and interaction Edge indicating both functional and physical protein associations (Figure 2J).

### Immune infiltration analysis associated with *GDI2*

**Figure 3:**
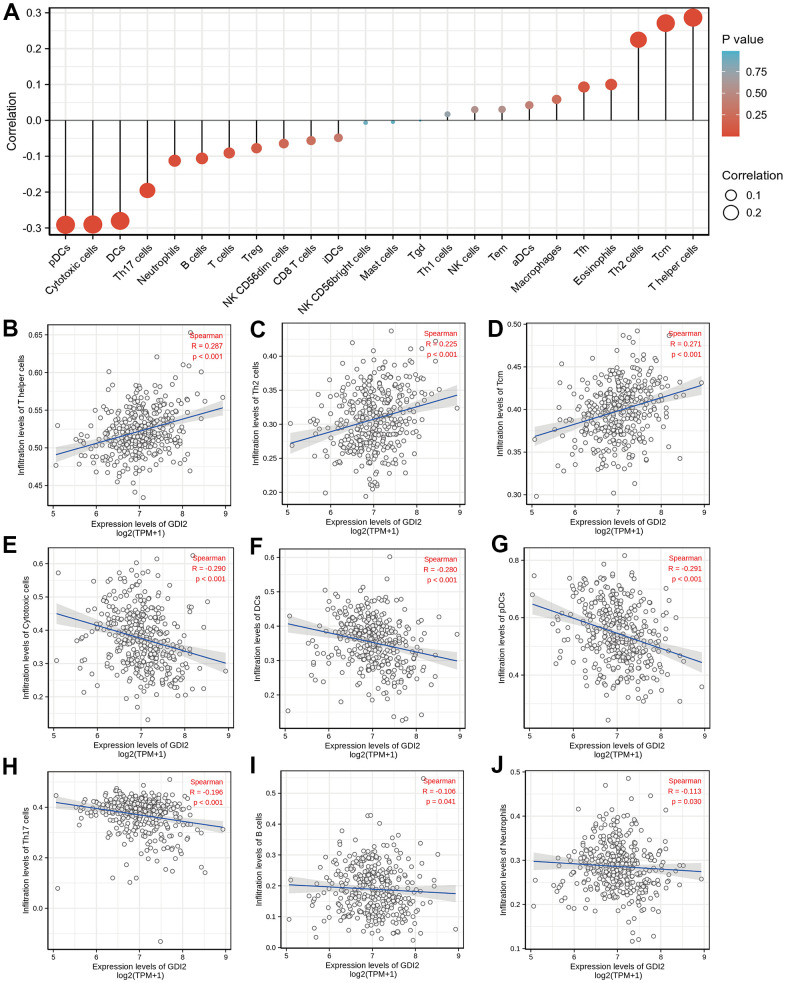
**Immune infiltration analysis based on *GDI2* expression by ssGSEA.** (**A**) The *GDI2* expression level was associated with the relative abundances of 24 immune cell subsets involved in immune infiltration in the tumor microenvironment. (**B**–**J**) Correlation between the relative enrichment score of immune cells and the expression level (TPM) of *GDI2*. The size of dots shows the absolute value of Spearman R. Positive correlations were found in (**B**) T helper cells; (**C**) Th2 cells; and (**D**) Tcm cells, Negative correlations were found in (**E**) Cytotoxic cells; (**F**) Dendritic cells (DCs); (**G**) Plasmacytoid Dendritic cells (pDCs); (**H**) Th17 cells; (**I**) B cells; and (**J**) Neutrophils. ^*^P < 0.05; |R| < 0.40.

The enrichment analyses indicated that the occurrence of HCC was strongly associated with tumor microenvironment (TME) like extracellular matrix (ECM) organization. Therefore, the relative tumor infiltration levels of a list of 509 genes were quantified and the abundance of a diverse set of 24 tumor-infiltrating adaptive and innate immune cell types was predicted in individual tissue sample by single-sample gene set enrichment analysis (ssGSEA) analysis (Figure 3A). Then, Spearman correlation analysis between *GDI2* expression and immune cell infiltration level displayed that the *GDI2* expression was positively correlated with the abundance of T helper cells (R = 0.287; *P* < 0.001; Figure 3B), Th2 cells (R = 0.225; *P* < 0.001; Figure 3C) and Tcm cells (R = 0.271; *P* < 0.001; Figure 3D); while negatively correlated with Cytotoxic cells (R = -0.290; *P* < 0.001; Figure 3E), Dendritic cells (DCs) (R = 0.-280; *P* < 0.001; Figure 3F), Plasmacytoid Dendritic cells (pDCs) (R = -0.291; *P* < 0.001; Figure 3G), Th17 cells (R = -0.196; *P* < 0.001; Figure 3H), B cells (R = -0.106; *P* = 0.041; Figure 3I), and Neutrophils (R = -0.113; *P* = 0.030; Figure 3J). While other immune cell subsets, including T cells, Treg cells, NK cells, and macrophages were weakly correlated with *GDI2* expression (Figure 3A).

### Association analyses of *GDI2* expression with clinicopathologic variables

**Figure 4:**
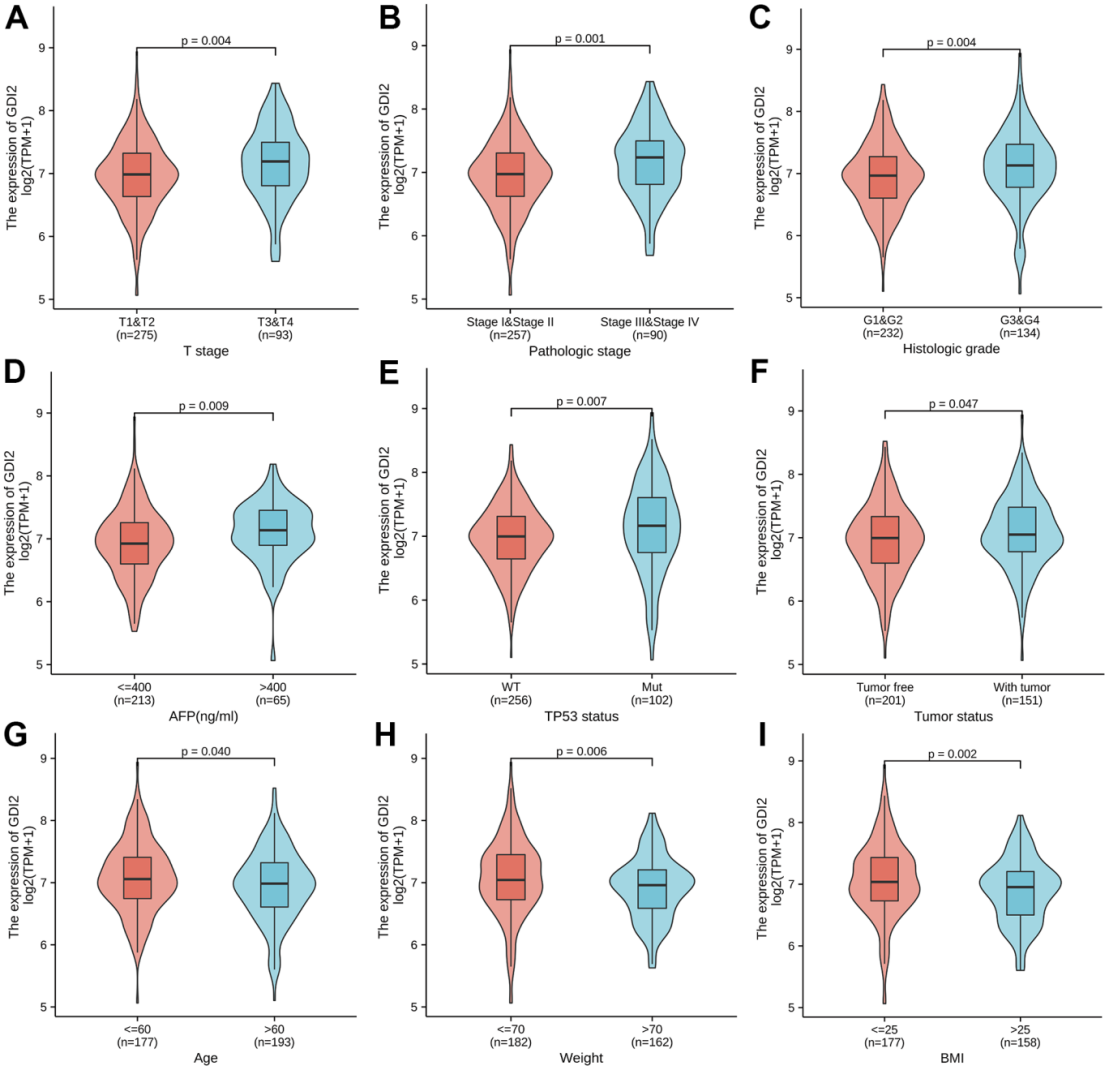
**Association between *GDI2* expression and clinical characteristics.** (**A**–**I**) Violin images for clinicopathologic characteristics as (**A**) T stage; (**B**) Pathologic stage; (**C**) Histologic grade, (**D**) AFP(ng/ml); (**E**) TP53 status; and (**F**) tumor status, as well as Physical index as (**G**) Age; (**H**) Weight; (**I**) BMI, demonstrated significant association with *GDI2* expression by Wilcoxon rank sum test. ^*^P < 0.05,^**^P < 0.01.

To explore the association between *GDI2* expression with 11 kinds of clinicopathologic characteristics, *GDI2* expression was firstly classified into high- and low-level, and Chi-square test was applied to explore the difference between high and low expression of *GDI2* in different clinicopathological characteristics of HCC patients (Table 1). Here, there was significant differences between high and low expressions of *GDI2* in HCC patients with clinical index as weight (*P* = 0.035), BMI (*P* = 0.020) and AFP (ng/ml) (*P* = 0.019). Then Logistics regression was applied to explore the clinical risky factors in HCC patients with *GDI2* expression on both TPM values and high- and low-classifications (Table 2). It was displayed that high- and low-expression of *GDI2* was only associated with histologic grade (OR=1.58(1.03-2.44); *P* = 0.035) (Table 2; upper); while consecutive TPM values of *GDI2* expression were associated with patients under more progressive stage (OR= 1.01(1.00-1.01) for T1 vs. T2/T3/T4, *P* < 0.001), more advanced pathologic stage (OR=1.01(1.00-1.01) for Stage I vs. Stage II/III/IV, *P* = 0.001), more serious histologic grade (OR=1.01(1.00-1.01) for G1/G2 vs. G3/G4, *P* = 0.007), and mutated TP53 status (OR=1.01(1.00-1.01) for WT vs. MUT, *P* < 0.001) (Table 2; lower). Though the odds ratios were critical (only 1.01(1.00-1.01)), it was indicated that the associations between *GDI2* and these four clinical factors were significant (*P < 0.05) but not strongly dependent. Finally, the Wilcoxon rank sum test was further applied and demonstrated that the *GDI2* expression was significantly associated with clinicopathologic features as T stage (T1-2 vs. T3-4; P = 0.004), Pathologic stage (PI-II vs. PIII-IV; *P* = 0.001), Histologic grade (G1-2 vs. G3-4; *P* = 0.004), AFP (ng/ml) level (AF*P*<=400 vs. >400; *P* = 0.009), TP53 status (WT vs. Mut; *P* = 0.007) and tumor status (Tumor free vs. With tumor; *P* = 0.047) (Figure 4A–4F); and consistently associated with personal physical index as Age (age<=60 vs. >60; *P* = 0.040), Weight (weight<=70 vs. >70; *P* = 0.006) and BMI (BMI<=25 vs. >25; *P* = 0.002) (Figure 4G–4I). Those association analyses suggested that *GDI2* expression was probably an independent risky factor for HCC patients, and significantly associated with clinicopathologic characteristics as T stage, pathologic stage, histologic grade and TP53 status.

**Table 2:** Association of clinicopathological characteristics with *GDI2* expression.

**Characteristics with high- and low-*GDI2* expression**	**Odds ratio (OR)**	***P* value**
T stage (T2&T3&T4 vs. T1)	1.45 (0.96-2.19)	0.076
N stage (N1 vs. N0)	2.60 (0.33-52.95)	0.411
M stage (M1 vs. M0)	0.94 (0.11-7.94)	0.952
Pathologic stage (Stage II&Stage III&Stage IV vs. Stage I)	1.40 (0.92-2.14)	0.119
Histologic grade (G3&G4 vs. G1&G2)	1.58 (1.03-2.44)	0.035^*^
Residual tumor (R1&R2 vs. R0)	1.36 (0.52-3.65)	0.525
Child-Pugh grade (B&C vs. A)	2.05 (0.84-5.31)	0.122
Fibrosis ishak score (1/2&3/4&5/6 vs. 0)	1.51 (0.86-2.69)	0.158
Adjacent hepatic tissue inflammation (Mild&Severe vs. None)	1.46 (0.87-2.45)	0.150
Vascular invasion (Yes vs. No)	1.18 (0.74-1.89)	0.475
Tumor status (With tumor vs. Tumor free)	1.25 (0.82-1.91)	0.303
TP53 status (Mut vs. WT)	1.51 (0.95-2.40)	0.083
**Characteristics with *GDI2* expression (TPM value)**	**Odds ratio (OR)**	***P* value**
T stage (T2&T3&T4 vs. T1)	1.01 (1.00-1.01)	<0.001^**^
N stage (N1 vs. N0)	1.01 (0.99-1.02)	0.309
M stage (M1 vs. M0)	1.00 (0.98-1.01)	0.755
Pathologic stage (Stage II&Stage III&Stage IV vs. Stage I)	1.01 (1.00-1.01)	0.001^**^
Histologic grade (G3&G4 vs. G1&G2)	1.01 (1.00-1.01)	0.007^**^
Residual tumor (R1&R2 vs. R0)	1.00 (1.00-1.01)	0.236
Child-Pugh grade (B&C vs. A)	1.01 (1.00-1.01)	0.130
Fibrosis ishak score (1/2&3/4&5/6 vs. 0)	1.00 (1.00-1.01)	0.112
Adjacent hepatic tissue inflammation (Mild&Severe vs. None)	1.01 (1.00-1.01)	0.051
Vascular invasion (Yes vs. No)	1.00 (1.00-1.01)	0.094
Tumor status (With tumor vs. Tumor free)	1.00 (1.00-1.01)	0.065
TP53 status (Mut vs. WT)	1.01 (1.00-1.01)	<0.001^**^

^*^P < 0.05; ^**^ *P* < 0.01.

### Prognostic analysis and model construction for *GDI2* expression in HCC patients

To explore the risky factors associated with patients’ prognosis, the Univariate Cox regression analysis was performed to figure out the hazard clinicopathologic indicators for HCC survival based on high- and low-*GDI2* expression, from which the significant variables (*P* < 0.1) as T stage (*P* < 0.001, hazard ratio (95% confidence interval) [HR (95%CI)] = 2.109 (1.469-3.028)), M stage (*P* = 0.018, HR (95%CI) = 4.032 (1.267-12.831)), pathologic stage (*P* < 0.001, HR (95%CI) = 2.074 (1.418-3.032)), tumor status (*P* < 0.001, HR (95%CI) = 2.361 (1.620-3.441)), and *GDI2* expression (*P* < 0.001, HR (95%CI) = 1.844 (1.298-2.620)), were put into the Multivariate Cox regression analysis for further investigation. The results confirmed that tumor status (*P* = 0.003, HR (95%CI) = 2.145 (1.300-3.539)) and *GDI2* (*P* = 0.014, HR (95%CI) = 1.836 (1.130-2.983)) were independent prognostic factors for HCC patients’ survival (*P < 0.05) (Table 3).

**Table 3:** Prognostic correlation between *GDI2* expression and clinical characteristics.

**Characteristics**	**Total (N)**	**HR (95% CI) univariate analysis**	***P* value univariate analysis**	**HR (95% CI) multivariate analysis**	***P* value multivariate analysis**
T stage (T2&T3&T4 vs. T1)	367	2.109 (1.469-3.028)	<0.001^**^	0.857 (0.116-6.338)	0.880
N stage (N1 vs. N0)	256	2.004 (0.491-8.181)	0.333		
M stage (M1 vs. M0)	270	4.032 (1.267-12.831)	**0.018**	1.774 (0.423-7.445)	0.433
Pathologic stage (Stage II&Stage III&Stage IV vs. Stage I)	346	2.074 (1.418-3.032)	<0.001^**^	2.652 (0.346-20.308)	0.348
Histologic grade (G3&G4 vs. G1&G2)	365	1.120 (0.781-1.606)	0.539		
Residual tumor (R1&R2 vs. R0)	341	1.571 (0.795-3.104)	0.194		
Age (>60 vs. <=60)	370	1.248 (0.880-1.768)	0.214		
Gender (Male vs. Female)	370	0.816 (0.573-1.163)	0.260		
Weight (>70 vs. <=70)	343	0.916 (0.640-1.312)	0.634		
Height (>=170 vs. < 170)	338	1.208 (0.833-1.753)	0.319		
BMI (>25 vs. <=25)	334	0.818 (0.563-1.186)	0.289		
Race (White vs. Asian&Black or African American)	358	1.245 (0.867-1.789)	0.235		
Child-Pugh grade (B&C vs. A)	238	1.616 (0.797-3.275)	0.183		
AFP(ng/ml) (>400 vs. <=400)	277	1.056 (0.646-1.727)	0.827		
Albumin(g/dl) (>=3.5 vs. <3.5)	296	0.921 (0.565-1.503)	0.743		
Prothrombin time (>4 vs. <=4)	293	1.330 (0.877-2.015)	0.179		
Fibrosis ishak score (1/2&3/4&5/6 vs. 0)	211	0.779 (0.470-1.293)	0.334		
Adjacent hepatic tissue inflammation (Mild&Severe vs. None)	233	1.228 (0.755-1.997)	0.409		
Vascular invasion (Yes vs. No)	314	1.348 (0.890-2.042)	0.159		
Tumor status (With tumor vs. Tumor free)	351	2.361 (1.620-3.441)	<0.001^**^	2.145 (1.300-3.539)	0.003^**^
TP53 status (Mut vs. WT)	357	1.434 (0.972-2.115)	0.069	1.602 (0.961-2.671)	0.071
*GDI2* (High vs. Low)	370	1.844 (1.298-2.620)	<0.001^**^	1.836 (1.130-2.983)	0.014^*^

^*^P < 0.05; ^**^ *P* < 0.01.

**Figure 5:**
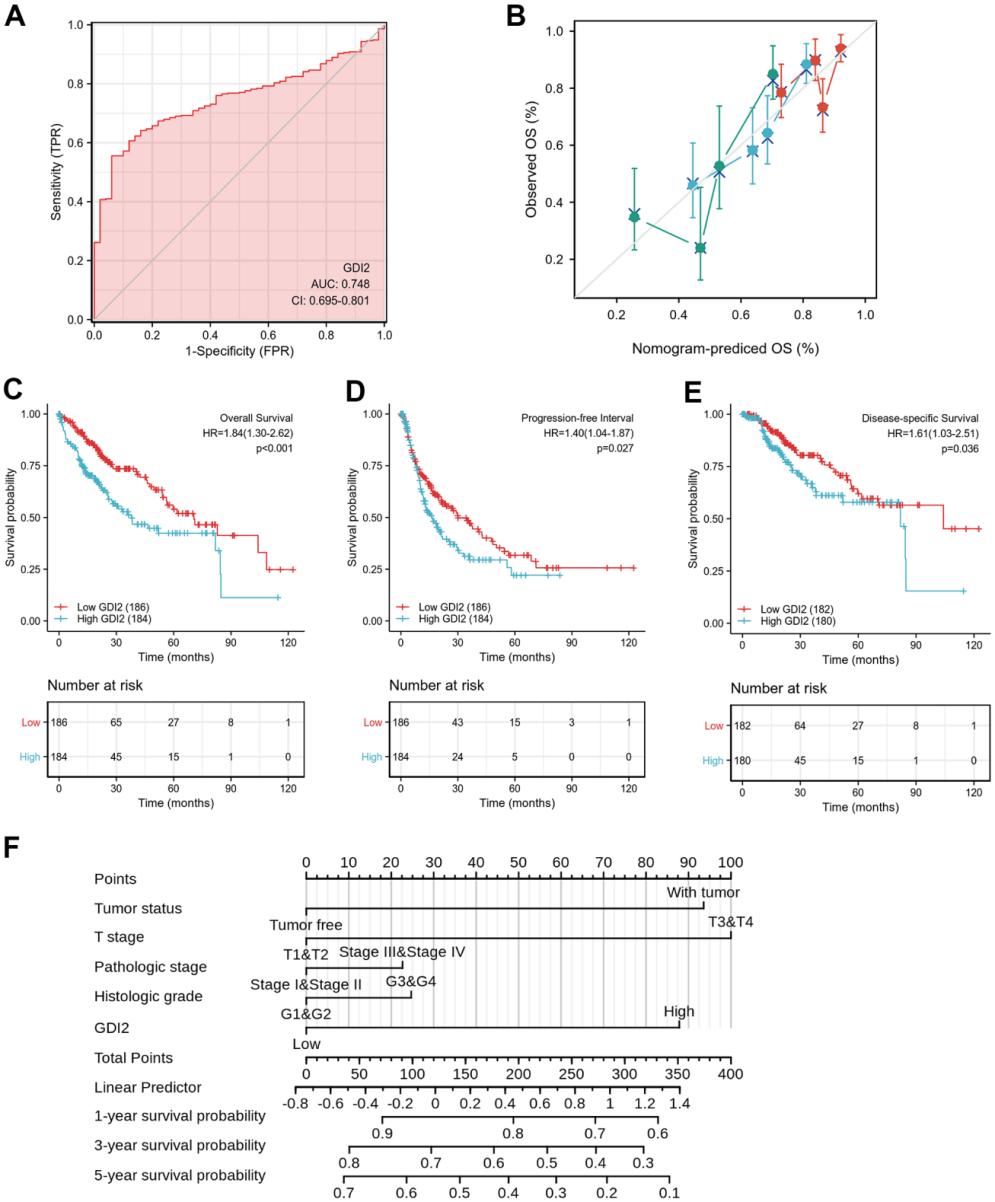
**Effective nomogram model for prognostic evaluation of *GDI2*.** (**A**) ROC analysis of *GDI2* showing a high ability to discriminate controls from liver samples validated in TCGA. The X-axis represents False Positive Rate (FPR), while the Y-axis denotes True Positive Rate (TPR). AUC is plotted as sensitivity% vs 100-specifificity%. (**B**) Nomogram to predict survival probability at 1, 2, and 3 years of OS for HCC patients. (**C**–**E**) High *GDI2* expression was associated with poor outcomes on (**C**) overall survival (OS), (**D**) progression-free interval (PFI), and (**E**) disease-specific survival (DSS) in HCC patients of a TCGA cohort. Blue: high *GDI2* (n=184); Red: low *GDI2* (n=186). ^*^ *P* < 0.05; ^**^ *P* < 0.01. (**F**) Calibration curve with Hosmer-Lemeshow test of the nomogram-predicted OS (%) in the TCGA-LIHC cohort relating to *GDI2* expression and tumor status, as well as T Stage, Pathologic Stage and Histologic Grade. The X-axis represents Prognostic Probability (0-100%), while the Y-axis denotes Observed OS (0-100%). Gray line: ideal line.

To validate whether the model construction was effective, receiver operating characteristic (ROC) curve was displayed to measure the discrimination value of *GDI2*. The calculated area under curve (AUC) of *GDI2* was 0.748, indicating that *GDI2* owned an efficient ability to discriminate hepatic carcinoma from normal liver and might be a potential diagnostic biomarker (Figure 5A). Then to provide clinicians with a quantitative approach to predicting the prognosis of HCC patients, a nomogram integrating *GDI2* and tumor status was constructed based on multivariate Cox analysis. We compared the predictive accuracy of this nomogram with that of *GDI2* and tumor status, obtaining the nomogram performance (C-index: 0.599 (0.573-0.626)) between predicted values 0.5 and 1.0. In addition, HCC patients with tumor (100 points) and high *GDI2* level (77 points) could receive a total point score of 177 in this nomogram. Then the probabilities of 1-, 3-, 5-year survival were determined by drawing a vertical line from the total point axis at a value of 177 straight downward to the outcome axis, respectively (Figure 5B).

Next, the Kaplan-Meier survival analysis was performed in 184 HCC patients with *GDI2*-high expression and 186 cases with *GDI2*-low expression to evaluate the relationship between *GDI2* expression and survival status of HCC patients in TCGA cohort. It showed that high *GDI2* expression was strongly associated with a worse overall survival (OS) (*P* < 0.001, HR (95%CI) = 1.84 (1.30-2.62); Figure 5C), a worse progression-free interval (PFI) (*P*=0.027, HR (95%CI) = 1.40 (1.04-1.87); Figure 5D), as well as a worse disease-specific survival (DSS) (*P* = 0.036, HR (95%CI) = 1.61 (1.03-2.51); Figure 5E) than that of low *GDI2* expression. In survival prediction nomogram for OS, the calibration curve conformed well to observations in all patients, integrating *GDI2* and tumor status index, as well as T stage, pathologic stage and histologic grade with a Hosmer-Lemeshow test, implied no departure from perfect fit (Figure 5F). These validations suggested that the models based on *GDI2* expression were effective for predicting short-term or long-term survival in HCC patients.

**Figure 6:**
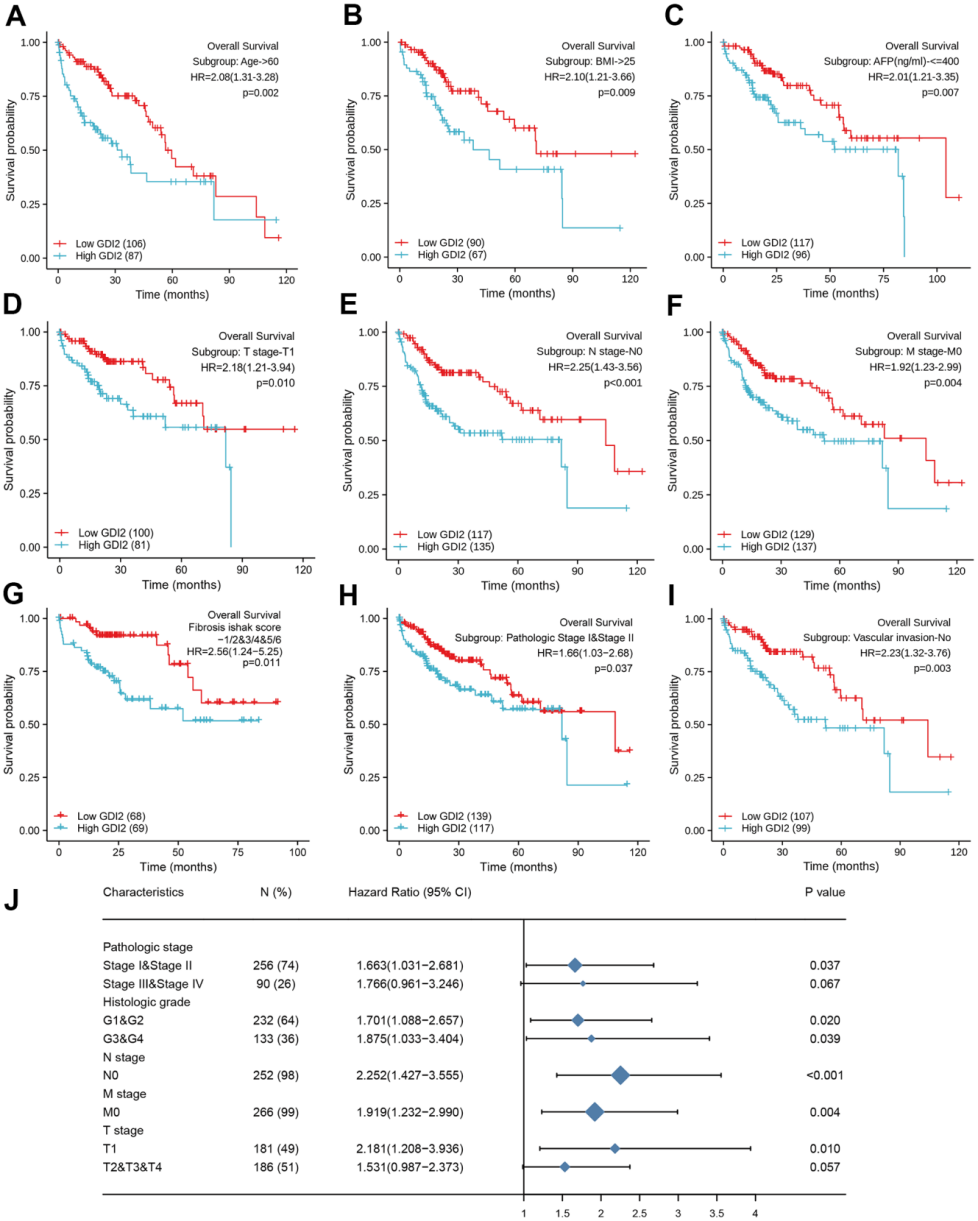
**Prognostic impact of clinical subtypes associated with *GDI2* expression in HCC patients.** (**A**–**I**) High *GDI2* expression was associated with poor outcomes on overall survival (OS) in HCC patients of a TCGA cohort with clinicopathological indicators as: (**A**) Age>60; (**B**) BMI>25; (**C**) AFP(ng/ml)<=400; (**D**) T1 Stage; (**E**) N0 Stage; (**F**) M0 Stage; (**G**) Fibrosis ishak score-1/2&3/4&5/6; (**H**) Pathologic Stage I&II; and (**I**) No Vascular invasion. Blue: high *GDI2*; Red: low *GDI2*. ^*^P < 0.05,^**^P < 0.01. (**J**) Forest map illustrated subtypes of clinicopathological features associated with *GDI2* expression for HCC prognosis. ^*^P < 0.05,^**^P < 0.01.

As for the subtypes of clinicopathological features associated with prognosis, the K-M survival analysis for sub-population displayed that *GDI2* expression significantly impacted the OS rate in HCC patients with certain clinical index as: Age>60 (HR=2.08(1.31-3.28); *P* = 0.002; Figure 6A), BMI>25 (HR=2.10(1.21-3.66); *P* = 0.009; Figure 6B), and AFP(ng/ml)<=400 (HR=2.01(1.21-3.35); *P* = 0.007; Figure 6C). Meanwhile, HCC patients with low *GDI2* expression could improve their prognosis when they were with clinicopathological subtypes as: T1 Stage (N=49, *P* = 0.010; HR (95%CI) = 2.18 (1.21-3.94); Figure 6D), negative lymph nodes (N=252, *P* < 0.001; HR (95%CI) = 2.252 (1.427-3.555); Figure 6E), without metastasis (N=266, *P* = 0.004; HR (95%CI) = 1.919 (1.232-2.990); Figure 6F), with fibrosis ishak score-1/2&3/4&5/6 (HR=2.56(1.24-5.25); *P*=0.011; Figure 6G); early pathologic stage I&II (HR=1.66(1.03-2.68); *P*=0.037; Figure 6H), and no vascular invasion (HR=2.23(1.32-3.76); *P*=0.003; Figure 6I). Then significant subtypes with prognostic impact were together intuitively illustrated by Forest Map (Figure 6J). These results suggested that *GDI2* could be applied as a prognostic indicator in early stage of HCC.

## DISCUSSION

Based on the specific bioinformatics analyses on *GDI2* based on The Cancer Genome Atlas (TCGA)-LIHC database, our results figured out that the *GDI2* significantly over-expressed in tumor tissues of HCC patients compared with normal tissues (Figure 1), acting as a tumor booster in HCC progression. Especially, in terms of that *GDI2* was positively correlated with the abundance of T helper cells, Tcm cells and Th2 cells, while negatively correlated with Cytotoxic cells and Dendritic cells (Figure 2), it could be speculated that the poor cytotoxic effect of immune cells might pave the way for tumor progression mediated by *GDI2*. As a result, high expression of *GDI2* turned out to be correlated with advanced tumor status (Figure 4) and poor prognosis (Figure 5).

As regulators of GDP-GTP exchange reaction of members of the Rab family, small GTP-binding proteins of the Ras superfamily, GDIs involve in many energy-related biological processes. There are two isoforms of Rab-GDIs, *GDI1* and *GDI2* genes. Anyway, it was reported that mutations in *GDI1* have been mainly linked to X-linked nonspecific cognitive disability [20], while more studies indicated that *GDI2* has been correlated with the energy-required tumors, yet leaving HCC uninvolved up to now. To be specific, the latest study in prostate cancer (PC) found that *GDI2* was a target of paclitaxel that affects tumorigenesis via p75NTR signaling pathway [21]. In human ovarian cancer (OC), paclitaxel-resistance [22] and tumor cell-induced fibroblasts were associated with *GDI2* up-regulation in OC cells [23]. While in breast cancer (BC), *GDI2* was found to contribute to EGFR endocytosis and thus enhance EGFR signaling and metastasis formation [15]. In human pancreatic adenocarcinoma (PAAD), *GDI2* was over expressed [16] and co-localized with Hsp90, together with family member Rab-GDI-1, they regulated agonist-induced amylase release in AR42J cells [24]. Likewise, *GDI2* was upregulated in anaplastic thyroid cancers (ATC) [25] and might be a genetic driver of metastatic dissemination in sonic hedgehog medulloblastoma [26]. Moreover, *GDI2* was differentially expressed in the secretome of esophageal squamous cell carcinoma (ESCC) [27] as well as in gastric cancer (GC) [17]. Gratifyingly, our findings were generally consistent with the reported studies mentioned above and showed that the *GDI2* gene was significantly over-expressed in most of the 33 types of cancers, such as lung adenocarcinoma (LUAD), Cholangiocarcinoma (CHOL), Breast Cancer (BRCA), Glioblastoma (GBM), endometrial carcinoma of uterus (UCEC), etc. In addition, our study explored for the first time that *GDI2* significantly over-expresses in tumor tissues of HCC patients compared with normal tissues, making it reasonable that *GDI2* could be considered as a tumor activator in future researches (Figure 1).

As for the *GDI2*-related pathways, the GO and KEGG, even GSEA enrichment analysis all indicated that *GDI2*-associated genes engaged in biological signaling mainly involving fatty acid metabolism and extracellular matrix (ECM) organization (Figure 2). This finding was generally consistent with Guzman-ruiz R’s study [28] that in obesity-associated insulin resistance (IR), *GDI2* altered lipid storage in adipocytes via dysregulation of both adipose tissue extracellular matrix organization and intracellular trafficking processes. Elsewhere, it was reported that 126kDa-*GDI2* interaction altered vesicle trafficking to enhance the establishment of a Tobacco mosaic virus (TMV) infection [29], which was accorded with transmembrane transporter enrichment. Considering that many HCC patients were originated from hepatitis B virus (HBV) infection, it could be assumed that *GDI2* might regulate the HBV-antigen vesicles trafficking to hepatocytes, similarly. Furthermore, since that HCC was mainly progressed from hepatic fibrosis (HF) till liver cirrhosis (LC), the *GDI2* might also participate in hepatic disease progression, similar to its role in multiple sclerosis (MS) [30]. Therefore, the *GDI2*-involved dominant pathways, not only Extracellular Structure Organization by GO and KEGG enrichment as BP_GO:0043062, MF_GO:0005201, CC_GO:0062023, and REACTOME_Cell extracellular matrix interactions by GSEA; but also ECM-related cell adhesion pathways, were of great instructive significance. Besides, as NAFLD is already the fastest growing cause of HCC globally [31–34], the lipid-related enrichments might put forward an inspiring direction for researchers to connect *GDI2*-mediates fatty acid metabolism with HCC. Notably, the GSEA analysis defined that the REACTOME_Rho GTPases Activate Formins (NES = 3.028, adj.*P* = 0.017, FDR = 0.009) pathway, and REACTOME_Immunoregulatory Interactions A Lymphoid and Non-lymphoid Cell (NES = 2.086, adj.*P* = 0.017, FDR = 0.009) pathway were also significantly enriched in our *GDI2* study (data not shown). In view of the immune infiltration analysis that *GDI2* expression was utmost positively correlated with T helper cells but utmost negatively with plasmacytoid Dendritic cells (pDC) (Figure 3), it could be speculated that *GDI2* might play an role in immune tumor microenvironment for HCC progression, which definitely requires profound and comprehensive researches for further confirmation.

In terms of physiological correlation, there were many biological functions associated with *GDI2* should be taken into consideration. For one thing, as the *GDI2* expression was related to Weight and BMI (**P < 0.01), energy-required and metabolism-related biological functions of *GDI2* could be emphasized. For another thing, the *GDI2* expression was significantly associated with the serum AFP(ng/ml) level and TP53 mutated status (**P < 0.01). Since that AFP is an indicator for HCC diagnosis, the co-expression of *GDI2* might tender it for a co-indicator in HCC diagnosis. While TP53, encoded by the typical antioncogene *P*53, is recently reported to be involved in the HIF1alpha/USP2/TP53 axis to promote hypoxia-induced HCC stemness [35], in the MiR-30e-3p/MDM2/TP53 axis to influence Sorafenib resistance in HCC [36], and a TP53-associated immune prognostic model for HCC has been developed and validated [37]. In view of that PID_*P*53 Regulation Pathway was enriched in *GDI2*-based GSEA analysis in our study (Figure 2H), the association of this novel protein *GDI2* with transcriptional factor TP53 should be attached great importance to, as well as their interactive mechanism in HCC tumorigenesis should be studied for further confirmation. As for the clinicopathological correlation for HCC patients, it was found that the expression of *GDI2* was increased as the disease progressed, especially for the HCC patients in late T stage, advanced pathologic stage, poor histologic grade, and with bad tumor status (*P < 0.05) (Figure 4). Since there was no reports demonstrating the clinicopathological correlation of *GDI2* with HCC, our study figured out that upregulation of *GDI2* might be applied as a diagnostic indicator for HCC progression for the first time.

What’s more, *GDI2* could also act as a prognostic biomarker to correlate HCC patients with high *GDI2* expression to poor prognosis, including poor overall survival (OS), poor progression-free interval (PFI), and poor disease-specific survival (DSS) (*P < 0.05) (Figure 5). On the contrary, HCC patients with low *GDI2* expression could strongly improve their OS rate especially when they were in clinicopathological subtypes as T1 Stage, pathologic Stage I/II, retarded fibrosis with negative lymph nodes, without metastasis and vascular invasion (*P < 0.05) (Figure 6).

Definitely, even though the multiple bioinformatic analyses in our study elucidate the significant correlation of *GDI2* gene with HCC for the first time, there were still a few limitations. Firstly, our results were mainly obtained from the TCGA database, lacking of comparisons among several databases made them not so comprehensive. Secondly, the sample size of HCC patients from TCGA with 371 cases was not large enough to acquire a convincing analytic result. Thirdly, the TCGA-LIHC samples of 371 patients were kinda of limited [38]: 1) There were three ethnic groups so that the genetic background and etiology of HCC among them could differ significantly; 2) The LIHC samples contain relatively few patients in T4 Stage, yet the clinical reality is that most HCC patients are with advanced disease and extremely poor prognosis when firstly diagnosed; 3) The transcriptome sequencing for RNAseq data can detect only static mutations at nucleic acid level, rather than directly provide information on expression or activity of proteins. Hence, follow-up studies and further confirmation should be carried out to fulfill these questions using molecular biology techniques. Anyway, considering that the clinical evaluation models, both Calibration discrimination and Nomogram evaluation were well-established; the results obtained in this study were basically creditable and instructive.

In conclusion, *GDI2* expression was explored to be elevated in HCC tumor tissues and associated with poor prognosis in HCC patients from our TCGA study for the first time. After further confirmation verifying the biological functions of *GDI2* and mechanisms of *GDI2*-related pathways, the relationship between *GDI2* and HCC could be fully elucidated. We believe that *GDI2* could be applied as a potential biomarker for diagnosis and prognosis for HCC patients, thus providing novel target and strategies for HCC treatment.

## MATERIALS AND METHODS

### Data collection for bioinformatics analysis from TCGA data repository

We downloaded level 3 HTSeq - FPKM formatted RNAseq data and clinical information from LIHC-hepatocellular carcinoma project from the website: https://portal.gdc.cancer.gov/ of The Cancer Genome Atlas (TCGA). A total of 371 cases with gene expression data and clinical information were collected by discarding those RNAseq data without clinical information [39]. Meanwhile, 50 normal control cases from GTEx database were downloaded from UCSC XENA (https://xenabrowser.net/datapages/) and unified handled by Toil procedure [40]. Level 3 RNAseq data formatted as HTSeq-FPKM (Fregments Per Kilobase per Million) from TCGA and GTEx were transformed into TPM (transcripts per million reads) for subsequent analyses. This study was in accordance with the publication guidelines provided by TCGA (https://cancergenome.nih.gov/publications/publicationguidelines). All data used in this study were obtained from TCGA without containing any human participants or animals performed by any of the authors, and hence ethics approval and informed consent were not required.

### Analysis of immune infiltration characteristics by ssGSEA

The ssGSEA (single-sample Gene Set Enrichment Analysis) method classifies marker gene sets in a single sample with common biological functions, chromosomal localization, and physiological regulation [41]. Normalized HCC gene expression profiles from formatted TPM data of single sample were compared with the immunocyte signatures using GSVA (R package) [42]. We quantified the relative tumor infiltration levels of immunocyte signature genes, including a total of 509 genes predicting the abundance of 24 tumor-infiltrating adaptive and innate immune cell types in individual tissue sample [43].

The following 24 types of immune cells were obtained: B cells; T cells, Helper T cells (Th), Cytotoxic T cells (Tc), CD4+ T cells, CD8+ T cells, type-1 T helper cells (Th1), type-2 T helper cells (Th2), type-17 T helper cells (Th17), Regulatory T cells (Treg), gamma delta T cells (γδT), central memory T cells (Tcm), effector memory T cells(Tem), follicular helper T cells(Tfh); Dendritic cells (DCs), activated Dendritic cells (aDCs), immature Dendritic cells (iDCs), plasmacytoid Dendritic cells (pDCs); natural killer (NK) cells, CD56 bright natural killer cellsr (CD56+NK), CD56 dim natural killer cells (CD56-NK); eosinophils, mast cells, neutrophils and macrophages. The correlation between *GDI2* and these immune cells was analyzed by Spearman correlation, and Wilcoxon rank sum test was adopted to explore the association of the infiltration levels of immune cells between the high- and low- expression groups of *GDI2* gene.

### DEGs analysis between high and low *GDI2* expression groups

We firstly used Wilcoxon Rank Sum Test to compare the expression of *GDI2* gene between tumor and normal specimens from TCGA combined with GTEx database in pan-cancers, referring to 33 kinds of solid tumors. Then in LIHC, *GDI2* expression was compared in both paired and unpaired normal-versus-tumor tissues. Finally, the *GDI2*expression profiles (HTSeq-counts) were compared between the divided high and low *GDI2* expression groups to identify differentially expressed genes (DEGs) by using DESeq2 R package [44]. |log2-fold change (FC)|>1 and adjusted P-value<0.05 were considered as threshold values for the DEGs.

### GO, KEGG and GSEA enrichment analysis

Gene Ontology (GO) functional analysis, including cellular component (CC), molecular function (MF), and biological process (BP), as well as Kyoto Encyclopedia of Genes and Genomes (KEGG) pathway analysis, were performed on the DEGs based on high and low *GDI2* expression levels by using the ClusterProfiler package (http://www.bioconductor.org/) [45] with P value adjusted by Benjamini and Hochberg method, from which adjusted P-value of <0.05 was considered as significance for GO function and KEGG pathway.

Gene Set Enrichment Analysis (GSEA; http://software.broadinstitute.org/gsea/index.jsp) is a computational method that determines whether prior defined functions or pathway sets of genes show statistical significance, concordant differences between two biological states [46]. Thus in this study, GSEA was applied to generate an ordered list of all genes according to their correlation with *GDI2* expression, then carried out by R package to elucidate the significant differences observed between high- and low- *GDI2* expression. The datasets h.all.v7.0.symbols.gmt [Hallmarks] (https://www.gsea-msigdb.org/gsea/msigdb/collections.jsp#H) and c2.cp.v7.0.symbols.gmt [Curated] (https://www.gsea-msigdb.org/gsea/msigdb/collections.jsp#C2) from MSigDB Collections were chosen as reference gene sets, and the expression level of *GDI2* was regarded as a phenotype. Gene set permutations were performed 1000 times for each analysis. A function or pathway term with adjusted P-value <0.05 and false discovery rate (FDR) <0.25 was considered to be statistically significant enrichment.

### Protein-protein interaction (PPI) network

Search Tool for the Retrieval of Interacting Genes (STRING; http://string-db.org) (version 11.0) online database was used to predict PPI network co-regulated by *GDI2* gene and exploring the functional interactions between co-regulatory proteins [47]. An interaction with a combined score > 0.7 was considered statistically significant.

### Clinical association and prognostic analysis

Wilcoxon rank sum test and t. test were utilized to evaluate the clinical index (age, gender, weight, BMI, AFP(ng/ml), etc.) in non-paired samples and paired samples with different expression levels of *GDI2* (the Fisher exact test was used when needed) [48]. The association of *GDI2* expression with clinicopathological features, such as TNM stage (TNM stage is a way of staging tumors, in which T represents the range of primary tumors, N represents the presence and extent of regional lymph node metastasis and its scope, M represents the presence or absence of a distant transfer), pathologic stage, histologic grade, and TP53 status, was evaluated by Chi-square test, and Logistics regression was performed on both continuous TPM values and high- and low-classifications of *GDI2* expression.

Clinical prognosis correlating *GDI2* to Overall Survival (OS), Progression-Free Interval (PFI) and Disease-Specific Survival (DSS) of HCC patients, was assessed using Survminer package [49]. Survival curves were constructed using the Kaplan-Meier method, and the differences between the survival curves were examined by the log-rank test [50]. Clinicopathological risky factors were evaluated by Cox regression analyses. Univariate Cox proportional hazards regressions were applied to estimate the individual hazard ratio (HR) with *GDI2* gene expression for HCC survival. The significant variables from the univariate analyses (*P* < 0.1) were then put into the multivariate analysis [51]. Multivariate Cox analysis was used to compare the influence of *GDI2* expression on survival along with other clinical characteristics in order to find independent variables. The HR with 95% confidence interval (CI) was measured to estimate the hazard risk of individual factors. Prognostic analyses were performed in both main-group and sub-group of clinicopathological indicators with *GDI2* expression, respectively.

### Nomogram evaluation

Calibration and discrimination are the most commonly used methods for evaluating the performance of models [52]. In this study, the Calibration curves were graphically assessed by mapping the nomogram-predicted probabilities against the observed rates, and the 45° line represented the best predictive values. A concordance index (C-index) was used to determine the discrimination of the nomogram, and it was calculated by a bootstrap approach with 1000 resamples [53]. The predictive accuracies of the nomogram and separate prognostic factors were compared using both the C-index and ROC (receiver operating characteristic) analysis. As the frequently-used method for binary assessment, ROC analysis was performed by pROC package [54] to assess the effectiveness of the transcriptional expression of *GDI2*. The computed area under the curve (AUC) value ranging from 0.5 to 1.0 indicates the discrimination ability from 50 to 100%.

### Cell lines and cell culture

The human normal hepatic cell line L02 and hepatic embryonic cell line WRL-68 were the kind gifts from Infection Department of First People’s Hospital of Yunnan Province. Seven human hepatoma cell lines: Huh7, SK-HEP1, BEL-7402, PLC/PRF/5, SMMC-7721, HepG2, Hep3B were purchased from Tongpai Biotechnology Company (Shanghai, China). All the cell lines were cultured in DMEM/high-glucose medium (Gibco, USA) supplemented with 10% fetal bovine serum (Gibco, USA) and 1% Penicillin-Streptomycin, and performed STR authentication [55].

### RNA isolation and qRT-PCR analysis

Total RNA from HCC cell lines was isolated using RNAprep FastPure Animal/Cell Total RNA Extraction Kit (TSINGKE, Beijing, China) according to manufacturer's protocols. Then approximately 2μg of RNA were reverse transcribed using the Goldenstar™ RT6 cDNA Synthesis kit (TSINGKE, Beijing, China). For *GDI2* detection, qRT-PCR labeled by SYBR green master mix (TSINGKE, Beijing, China) was performed on a LightCycler 480 system (Roche, USA). To confirm the expression from different transcripts of *GDI2*, primers indicating different transcripts and common primers of *GDI2* for qRT-PCR are listed as below: *GDI2*: Forward: 5’ATTCCACAGAACCAAGTCAATCGA 3’; Reverse: 5’CTTCTCAGGCTCCTTGGTTTCC 3’; *GDI2*-Transcript I: Forward: 5'GGGCACCGGCCTGACGGAATGTA 3'; Reverse: 5'TGCCAGGGCTTCTGCTTCAGTGG 3'; *GDI2*-Transcript II: Forward: 5'CCACCCGAGTCAATGGGGAGAGG 3'; Reverse: 5'CTTGATGGGGTGGCTGAGGATGC 3'. ACTB was used as an internal reference: Forward: 5’CACCATTGGCAATGAGCGGTTCA 3’; Reverse: 5’AGGTCTTTGCGGATGTCCACGT 3’.

### Western blot

Cultured cells were collected and washed twice with ice-cold PBS, then lysed in RIPA lysis mixed with PSMF (Beyotime, China) buffer on ice for 30 minutes. Cell lysates were collected immediately according to the manufacturer’s instructions and subjected to bicinchoninic acid (BCA) protein assay (Beyotime, China) for concentration determination. Equal amounts of proteins in each lane were separated by SDS-PAGE and subsequently transferred onto polyvinylidene fluoride (PVDF) membranes (Millipore, USA). The membranes were blocked with 3% (w/v) skim milk and then incubated with specific primary antibody at dilution of 1:5000 (anti-*GDI2*; Invitrogen, USA) overnight followed by incubation with horseradish peroxidase (HRP)-conjugated secondary antibody at dilution of 1:10000 (anti-rabbit-HRP; Cell Signaling Technology, USA). β-actin was used as an internal control at dilution of 1:10000 (anti-beta Actin; Cell Signaling Technology, USA). An enhanced chemiluminescence (ECL) chemiluminescence kit (ABclonal, China) was used to detect immunoreactive protein bands using a Gel Doc XR imaging system (Bio-Rad, USA).

### Statistical analysis

All statistical analysis and plots referring to phenotype and expression profiles in 371 HCC patients from TCGA-LIHC were conducted using R package (v.3.5.1) (http://cran.r-project.org/web/packages/rms/index.html). In gene expression analysis, the median GDI2 expression was regarded as the cut-off value. All hypothetical tests were two-tailed and all reported *P* values < 0.05 were considered significant, marked as *P< 0.05; **P < 0.01. The statistical analysis was carried out using Graphpad Prism 8.0 and SPSS version 22.0.
